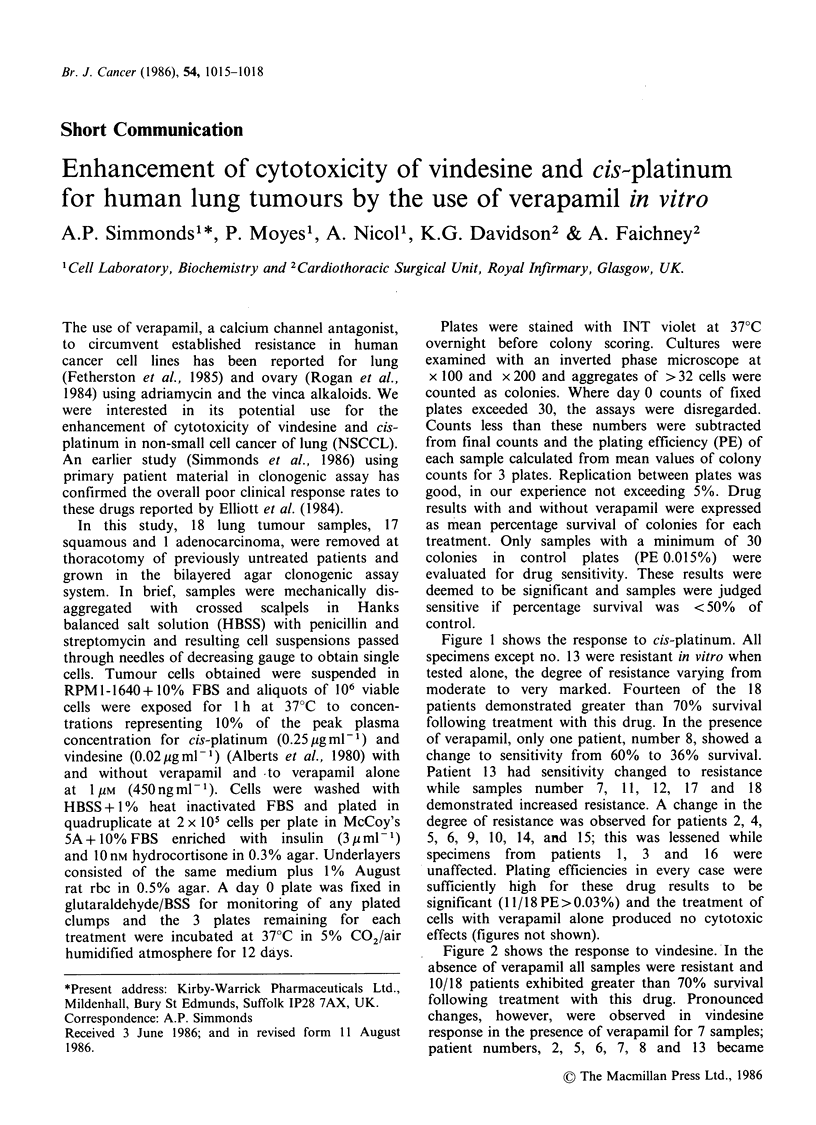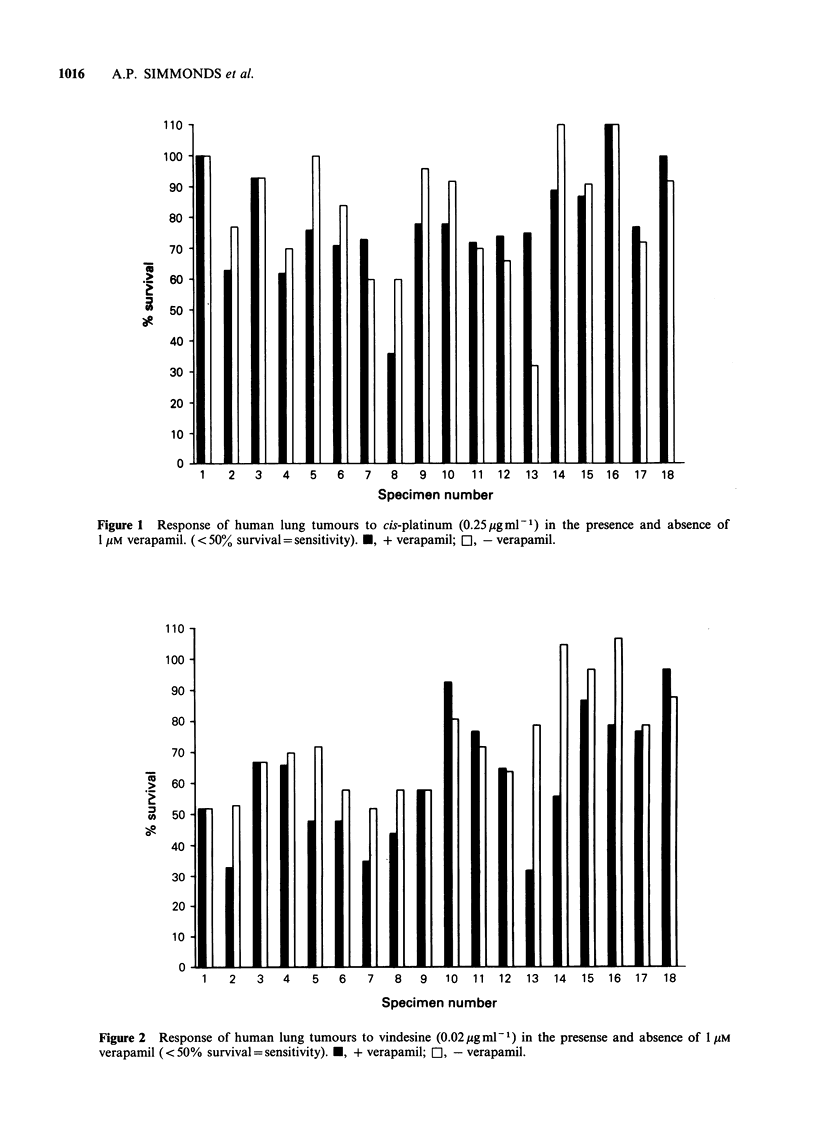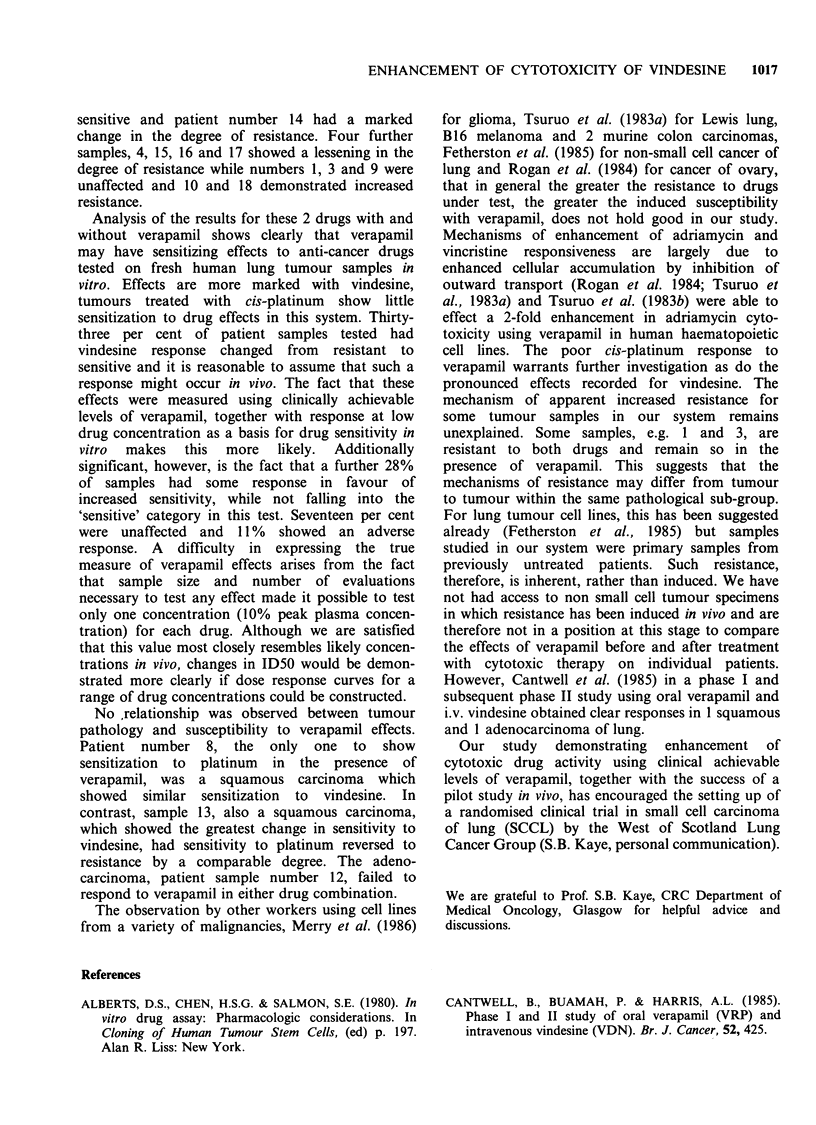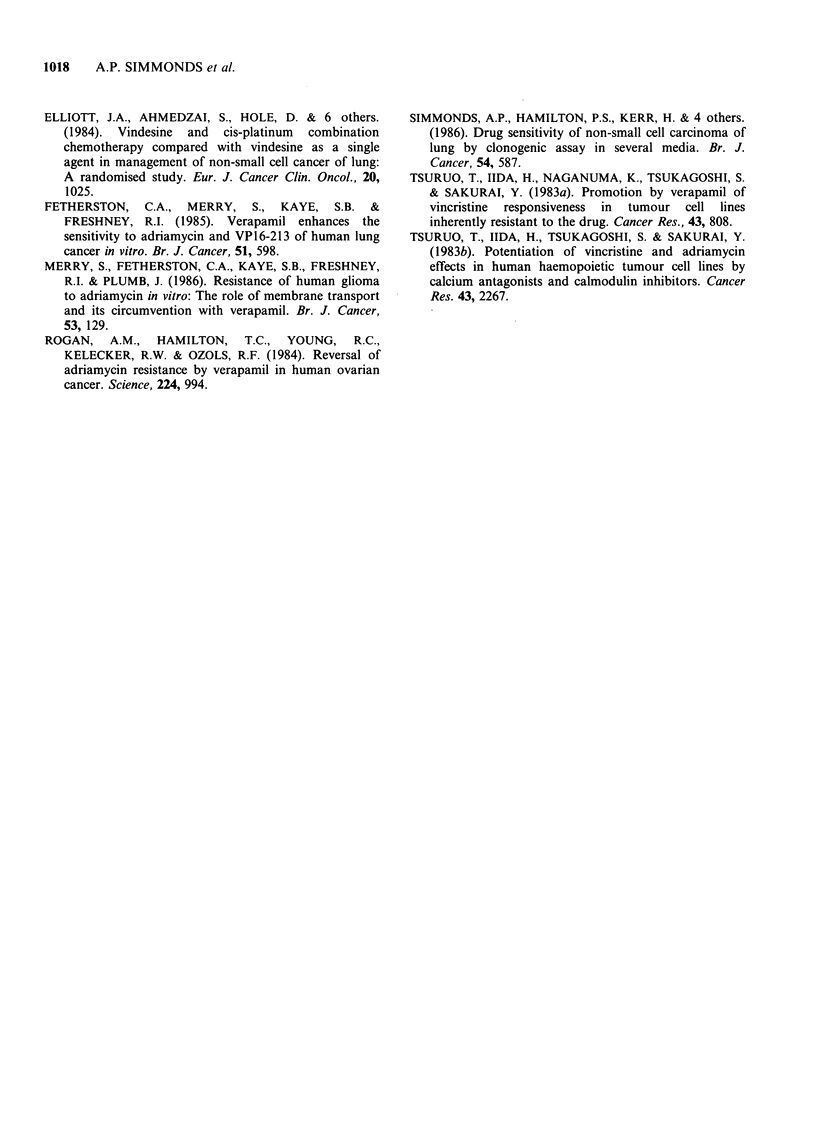# Enhancement of cytotoxicity of vindesine and cis-platinum for human lung tumours by the use of verapamil in vitro.

**DOI:** 10.1038/bjc.1986.274

**Published:** 1986-12

**Authors:** A. P. Simmonds, P. Moyes, A. Nicol, K. G. Davidson, A. Faichney


Br. J. Cancer (1986), 54, 1015–1018

### Short Communication

### Enhancement of cytotoxicity of vindesine and *cis*-platinum for human lung tumours by the use of verapamil *in vitro*

A.P. Simmonds[1]*, P. Moyes[1], A. Nicol[1], K.G. Davidson[2] & A. Faichney[2]

[1]*Cell Laboratory, Biochemistry and* [2]*Cardiothoracic Surgical Unit, Royal Infirmary, Glasgow, UK.*

The use of verapamil, a calcium channel antagonist, to circumvent established resistance in human cancer cell lines has been reported for lung (Fetherston *et al.*, 1985) and ovary (Rogan *et al.*, 1984) using adriamycin and the vinca alkaloids. We were interested in its potential use for the enhancement of cytotoxicity of vindesine and *cis*-platinum in non-small cell cancer of lung (NSCCL). An earlier study (Simmonds *et al.*, 1986) using primary patient material in clonogenic assay has confirmed the overall poor clinical response rates to these drugs reported by Elliott *et al.* (1984).

In this study, 18 lung tumour samples, 17 squamous and 1 adenocarcinoma, were removed at thoracotomy of previously untreated patients and grown in the bilayered agar clonogenic assay system. In brief, samples were mechanically disaggregated with crossed scalpels in Hanks balanced salt solution (HBSS) with penicillin and streptomycin and resulting cell suspensions passed through needles of decreasing gauge to obtain single cells. Tumour cells obtained were suspended in RPM1-1640+10% FBS and aliquots of $10^6$ viable cells were exposed for 1 h at 37°C to concentrations representing 10% of the peak plasma concentration for *cis*-platinum ($0.25\,\mu g\,ml^{-1}$) and vindesine ($0.02\,\mu g\,ml^{-1}$) (Alberts *et al.*, 1980) with and without verapamil and to verapamil alone at $1\,\mu$M ($450\,ng\,ml^{-1}$). Cells were washed with HBSS+1% heat inactivated FBS and plated in quadruplicate at $2\times10^5$ cells per plate in McCoy's 5A+10% FBS enriched with insulin ($3\,\mu ml^{-1}$) and 10 nM hydrocortisone in 0.3% agar. Underlayers consisted of the same medium plus 1% August rat rbc in 0.5% agar. A day 0 plate was fixed in glutaraldehyde/BSS for monitoring of any plated clumps and the 3 plates remaining for each treatment were incubated at 37°C in 5% $CO_2$/air humidified atmosphere for 12 days.

Plates were stained with INT violet at 37°C overnight before colony scoring. Cultures were examined with an inverted phase microscope at ×100 and ×200 and aggregates of >32 cells were counted as colonies. Where day 0 counts of fixed plates exceeded 30, the assays were disregarded. Counts less than these numbers were subtracted from final counts and the plating efficiency (PE) of each sample calculated from mean values of colony counts for 3 plates. Replication between plates was good, in our experience not exceeding 5%. Drug results with and without verapamil were expressed as mean percentage survival of colonies for each treatment. Only samples with a minimum of 30 colonies in control plates (PE 0.015%) were evaluated for drug sensitivity. These results were deemed to be significant and samples were judged sensitive if percentage survival was <50% of control.

Figure 1 shows the response to *cis*-platinum. All specimens except no. 13 were resistant *in vitro* when tested alone, the degree of resistance varying from moderate to very marked. Fourteen of the 18 patients demonstrated greater than 70% survival following treatment with this drug. In the presence of verapamil, only one patient, number 8, showed a change to sensitivity from 60% to 36% survival. Patient 13 had sensitivity changed to resistance while samples number 7, 11, 12, 17 and 18 demonstrated increased resistance. A change in the degree of resistance was observed for patients 2, 4, 5, 6, 9, 10, 14, and 15; this was lessened while specimens from patients 1, 3 and 16 were unaffected. Plating efficiencies in every case were sufficiently high for these drug results to be significant (11/18 PE>0.03%) and the treatment of cells with verapamil alone produced no cytotoxic effects (figures not shown).

Figure 2 shows the response to vindesine. In the absence of verapamil all samples were resistant and 10/18 patients exhibited greater than 70% survival following treatment with this drug. Pronounced changes, however, were observed in vindesine response in the presence of verapamil for 7 samples; patient numbers, 2, 5, 6, 7, 8 and 13 became

*Present address: Kirby-Warrick Pharmaceuticals Ltd., Mildenhall, Bury St Edmunds, Suffolk IP28 7AX, UK.
Correspondence: A.P. Simmonds
Received 3 June 1986; and in revised form 11 August 1986.

© The Macmillan Press Ltd., 1986

**Figure 1** Response of human lung tumours to *cis*-platinum (0.25 μg ml$^{-1}$) in the presence and absence of 1 μM verapamil. (<50% survival = sensitivity). ■, + verapamil; □, − verapamil.

**Figure 2** Response of human lung tumours to vindesine (0.02 μg ml$^{-1}$) in the presense and absence of 1 μM verapamil (<50% survival = sensitivity). ■, + verapamil; □, − verapamil.

sensitive and patient number 14 had a marked change in the degree of resistance. Four further samples, 4, 15, 16 and 17 showed a lessening in the degree of resistance while numbers 1, 3 and 9 were unaffected and 10 and 18 demonstrated increased resistance.

Analysis of the results for these 2 drugs with and without verapamil shows clearly that verapamil may have sensitizing effects to anti-cancer drugs tested on fresh human lung tumour samples *in vitro*. Effects are more marked with vindesine, tumours treated with *cis*-platinum show little sensitization to drug effects in this system. Thirty-three per cent of patient samples tested had vindesine response changed from resistant to sensitive and it is reasonable to assume that such a response might occur *in vivo*. The fact that these effects were measured using clinically achievable levels of verapamil, together with response at low drug concentration as a basis for drug sensitivity *in vitro* makes this more likely. Additionally significant, however, is the fact that a further 28% of samples had some response in favour of increased sensitivity, while not falling into the 'sensitive' category in this test. Seventeen per cent were unaffected and 11% showed an adverse response. A difficulty in expressing the true measure of verapamil effects arises from the fact that sample size and number of evaluations necessary to test any effect made it possible to test only one concentration (10% peak plasma concentration) for each drug. Although we are satisfied that this value most closely resembles likely concentrations *in vivo*, changes in ID50 would be demonstrated more clearly if dose response curves for a range of drug concentrations could be constructed.

No relationship was observed between tumour pathology and susceptibility to verapamil effects. Patient number 8, the only one to show sensitization to platinum in the presence of verapamil, was a squamous carcinoma which showed similar sensitization to vindesine. In contrast, sample 13, also a squamous carcinoma, which showed the greatest change in sensitivity to vindesine, had sensitivity to platinum reversed to resistance by a comparable degree. The adenocarcinoma, patient sample number 12, failed to respond to verapamil in either drug combination.

The observation by other workers using cell lines from a variety of malignancies, Merry *et al.* (1986) for glioma, Tsuruo *et al.* (1983*a*) for Lewis lung, B16 melanoma and 2 murine colon carcinomas, Fetherston *et al.* (1985) for non-small cell cancer of lung and Rogan *et al.* (1984) for cancer of ovary, that in general the greater the resistance to drugs under test, the greater the induced susceptibility with verapamil, does not hold good in our study. Mechanisms of enhancement of adriamycin and vincristine responsiveness are largely due to enhanced cellular accumulation by inhibition of outward transport (Rogan *et al.* 1984; Tsuruo *et al.*, 1983*a*) and Tsuruo *et al.* (1983*b*) were able to effect a 2-fold enhancement in adriamycin cytotoxicity using verapamil in human haematopoietic cell lines. The poor *cis*-platinum response to verapamil warrants further investigation as do the pronounced effects recorded for vindesine. The mechanism of apparent increased resistance for some tumour samples in our system remains unexplained. Some samples, e.g. 1 and 3, are resistant to both drugs and remain so in the presence of verapamil. This suggests that the mechanisms of resistance may differ from tumour to tumour within the same pathological sub-group. For lung tumour cell lines, this has been suggested already (Fetherston *et al.*, 1985) but samples studied in our system were primary samples from previously untreated patients. Such resistance, therefore, is inherent, rather than induced. We have not had access to non small cell tumour specimens in which resistance has been induced *in vivo* and are therefore not in a position at this stage to compare the effects of verapamil before and after treatment with cytotoxic therapy on individual patients. However, Cantwell *et al.* (1985) in a phase I and subsequent phase II study using oral verapamil and i.v. vindesine obtained clear responses in 1 squamous and 1 adenocarcinoma of lung.

Our study demonstrating enhancement of cytotoxic drug activity using clinical achievable levels of verapamil, together with the success of a pilot study *in vivo*, has encouraged the setting up of a randomised clinical trial in small cell carcinoma of lung (SCCL) by the West of Scotland Lung Cancer Group (S.B. Kaye, personal communication).

We are grateful to Prof. S.B. Kaye, CRC Department of Medical Oncology, Glasgow for helpful advice and discussions.

### References

ALBERTS, D.S., CHEN, H.S.G. & SALMON, S.E. (1980). *In vitro* drug assay: Pharmacologic considerations. In *Cloning of Human Tumour Stem Cells*, (ed) p. 197. Alan R. Liss: New York.

CANTWELL, B., BUAMAH, P. & HARRIS, A.L. (1985). Phase I and II study of oral verapamil (VRP) and intravenous vindesine (VDN). *Br. J. Cancer*, **52**, 425.

ELLIOTT, J.A., AHMEDZAI, S., HOLE, D. & 6 others. (1984). Vindesine and cis-platinum combination chemotherapy compared with vindesine as a single agent in management of non-small cell cancer of lung: A randomised study. *Eur. J. Cancer Clin. Oncol.*, **20,** 1025.

FETHERSTON, C.A., MERRY, S., KAYE, S.B. & FRESHNEY, R.I. (1985). Verapamil enhances the sensitivity to adriamycin and VP16-213 of human lung cancer *in vitro*. *Br. J. Cancer*, **51,** 598.

MERRY, S., FETHERSTON, C.A., KAYE, S.B., FRESHNEY, R.I. & PLUMB, J. (1986). Resistance of human glioma to adriamycin *in vitro*: The role of membrane transport and its circumvention with verapamil. *Br. J. Cancer*, **53,** 129.

ROGAN, A.M., HAMILTON, T.C., YOUNG, R.C., KELECKER, R.W. & OZOLS, R.F. (1984). Reversal of adriamycin resistance by verapamil in human ovarian cancer. *Science*, **224,** 994.

SIMMONDS, A.P., HAMILTON, P.S., KERR, H. & 4 others. (1986). Drug sensitivity of non-small cell carcinoma of lung by clonogenic assay in several media. *Br. J. Cancer*, **54,** 587.

TSURUO, T., IIDA, H., NAGANUMA, K., TSUKAGOSHI, S. & SAKURAI, Y. (1983*a*). Promotion by verapamil of vincristine responsiveness in tumour cell lines inherently resistant to the drug. *Cancer Res.*, **43,** 808.

TSURUO, T., IIDA, H., TSUKAGOSHI, S. & SAKURAI, Y. (1983*b*). Potentiation of vincristine and adriamycin effects in human haemopoietic tumour cell lines by calcium antagonists and calmodulin inhibitors. *Cancer Res.* **43,** 2267.